# Randomized Controlled Trial of *Game Changers*, a Social Network Intervention for HIV Prevention in Uganda

**DOI:** 10.1007/s10461-025-04907-4

**Published:** 2025-11-11

**Authors:** Laura M. Bogart, Joseph K. B. Matovu, Harold D. Green, Susan Ninsiima, David J. Klein, Violet Gwokyalya, Richard Serunkuuma, Bonnie Ghosh-Dastidar, Kuraish Mubiru, Milly Nabukeera, Nipher Malika, Stephen Okoboi, Glenn J. Wagner

**Affiliations:** 1 RAND, 1776 Main Street, P.O. Box 2138, Santa Monica, CA 90407-2138 USA; 2 Charles R. Drew University of Medicine and Science, Los Angeles, CA USA; 3 Makerere University School of Public Health, Kampala, Uganda; 4 Busitema University Faculty of Health Sciences, Mbale, Uganda; 5 Indiana University School of Public Health—Bloomington, Bloomington, IN USA; 6 Infectious Diseases Institute, Kampala, Uganda; 7 National Forum of People Living With HIV/AIDS Networks in Uganda (NAFOPHANU), Kampala, Uganda; 8 The Positive Men’s Union (POMU), Kampala, Uganda; 9 Uganda Young Positives, Kampala, Uganda

**Keywords:** HIV, Intervention, Prevention advocacy, Randomized controlled trial, Social network, Stigma

## Abstract

In Uganda, where HIV prevalence remains above 5% among those aged 15 and older, we conducted a randomized controlled trial of *Game Changers*, an 8-session peer-facilitated group intervention that empowers people living with HIV (PLHIV) to engage in HIV prevention advocacy with their social network members (“alters”). A total of 210 index PLHIV participants (105 intervention, 105 control) and 599 alters were enrolled; assessments were conducted at baseline and 6-, 12-, and 18-months post-baseline. Intention-to-treat repeated measures regressions indicated significant intervention effects on reduced internalized stigma and increased HIV prevention advocacy, prevention advocacy self-efficacy, and disclosure among index participants. In within-intervention group analyses, alters exposed to prevention advocacy showed higher likelihoods of HIV testing and condom use. *Game Changers* had direct psychosocial benefits for index participants, and indirect benefits for improved HIV prevention behaviors among alters. Implementation research is needed to determine conditions under which *Game Changers* is most effective.

NCT05098015, Registered 2021-10-18.

## Resumen

En Uganda, donde la prevalencia del VIH se mantiene por encima del 5% entre las personas de 15 años o más, llevamos a cabo un ensayo controlado aleatorio de *Game Changers*, una intervención grupal facilitada por pares de 8 sesiones que empodera a las personas con VIH para que participen en la promoción de la prevención del VIH con los miembros de su red social (“alter”). Se inscribieron un total de 210 participantes (105 en el grupo de intervención, y 105 en el grupo de control) y 599 alters; se realizaron evaluaciones al inicio del estudio y a los 6, 12 y 18 meses después del inicio. Las regresiones de medidas repetidas por intención de tratar indicaron efectos significativos de la intervención en la reducción del estigma internalizado y el aumento de la promoción de la prevención del VIH, la autoeficacia en la promoción de la prevención y la divulgación entre los participantes del índice. En los análisis realizados dentro del grupo de intervención, los alters expuestos a la promoción de la prevención mostraron mayores probabilidades de hacerse la prueba del VIH y de utilizar condones. *Game Changers* tuvo beneficios psicosociales directos para los participantes índice y beneficios indirectos para mejorar los comportamientos de prevención del VIH entre los alters. Es necesario realizar investigaciones sobre la implementación para determinar las condiciones bajo las cuales *Game Changers* es más efectivo.

## Introduction

High HIV rates persist in sub-Saharan Africa, including in Uganda, where an estimated 5.8% of people aged 15 and older are living with HIV [1]. Relatively small percentages of the population at increased risk for HIV acquisition use condoms and pre-exposure prophylaxis (PrEP; < 3% estimated, of those eligible) [2]—and PrEP use is likely to plummet, and HIV incidence is likely to increase, unless restrictions on funding from the US President’s Emergency Plan for AIDS Relief (PEPFAR) are lifted [3]. Continued emphasis on HIV prevention, including messages to encourage HIV testing and condom use, is needed to control the HIV epidemic.

In Uganda and globally, HIV stigma is a key driver of behaviors, such as condomless sex, that lead to HIV transmission [4]. In the 2022 Uganda Demographic and Health Survey [5], about a quarter of adults aged 15–49 endorsed stigmatizing beliefs about HIV (i.e., that they would not buy vegetables from a vendor with HIV, and did not think children with HIV should attend school with HIV-negative children). Research suggests that such beliefs weaken prevention efforts. For example, people who perceive high levels of HIV stigma in their social networks may avoid HIV testing for fear of testing positive and being discriminated against (i.e., anticipated stigma such as gossip, rejection, and violence) [6–8]. PLHIV who internalize stigma (agree with negative HIV-related stereotypes) may not disclose their status, not use or adhere to ART (for fear of others seeing their medications), not use condoms consistently, or not seek HIV care [9–12]. Social support can buffer against stigma by providing information to counteract misconceptions; tangible assistance (e.g., reminders) for HIV testing, adherence, and care-seeking; and emotional comfort that improves adaptive coping and empowers HIV testing and ART adherence [13–15]. Given the key role of social support in overcoming stigma, some anti-stigma interventions have involved social network members (e.g., family, caregivers)—but few have targeted social networks as a whole [16, 17].

One promising method for leveraging social support and disseminating HIV information is to empower PLHIV to act as change agents for HIV prevention in their social networks. Research suggests that many PLHIV, especially those with lower internalized stigma who are successfully managing their own health, are motivated to protect family and friends and educate them about HIV prevention, including the benefits of condom use, HIV treatment, and HIV testing [18, 19]. Although PLHIV may lack the skills to convey prevention advocacy messages effectively enough to lead to social network members’ behavior change, they can be trained in evidence-based behavioral skills for effective communication in dyadic relationships, including for prevention advocacy [20–22]. Moreover, PLHIV are likely to be perceived as credible and trustworthy sources of HIV information by social network members, because they have direct experience of living positively with HIV and are motivated by caring and empathy for close family members and friends who typically trust and respect them.

We conducted a randomized controlled trial of *Game Changers for HIV Prevention* (“*Game Changers*”), an 8-session group social network-based intervention that aims to increase HIV testing and condom use among social network members of PLHIV. *Game Changers* includes content for PLHIV “index” participants on internalized HIV stigma, safe serostatus disclosure, and skills-building on communication skills for prevention advocacy—in order to empower PLHIV to advocate with social network members (“alters”). *Game Changers* adds to the body of intervention research on HIV testing that leverages social networks [23] and HIV stigma reduction in low and middle income countries [24]. Social diffusion and social influence theories, which posit that a subgroup of key, credible individuals can effectively spread information and accelerate changes in social and behavioral norms through social networks [25–29], guide the *Game Changers* model, in which PLHIV are credible sources who can influence social network members through the dissemination of information about HIV, which in turn, can result in improved HIV prevention behaviors. In addition, *Game Changers* aims to increase knowledge and awareness around HIV in social networks, strategies that have been shown to decrease fear, shame, stigmatizing beliefs and discrimination, and in turn, increase HIV testing in low and middle income countries [24].

By focusing on PLHIV as agents of change within social networks, *Game Changers* extends prior social network intervention research in HIV prevention, which has enlisted popular opinion leaders regardless of serostatus or groups of peers without HIV to spread information [20, 21, 30]. For example, the evidence-based Peer Change Agent and Popular Opinion Leader (POL) models have been used to activate members of a specific community or risk group to be agents for prevention and advocate to other members of the same group [20, 31–33]. However, no existing program empowers and trains PLHIV to engage in advocacy to do prevention advocacy widely, across all members of their social network.

A pilot randomized controlled trial of *Game Changers* showed effects on increased HIV prevention advocacy, reduced internalized HIV stigma, and increased disclosure among index PLHIV participants, and marginally reduced condom [33] less sex among their social network members [34]. However, this small pilot was not statistically powered to test the intervention’s effects on PLHIV’s social network members. In the present fully powered randomized controlled trial, we hypothesized that *Game Changers* would lead to increased HIV prevention advocacy, prevention advocacy self-efficacy, HIV knowledge, and status disclosure, and reduced internalized HIV stigma, among index PLHIV participants, and increased HIV testing and consistent condom use among their social network members. By harnessing PLHIV’s natural tendency for advocacy in a high prevalence setting, *Game Changers* holds promise for widespread penetration of HIV prevention messaging, and consequent behavior change, in the country.

## Methods

### Study Design

Full details of the study methods, intervention, and assessment are provided in a prior article [35]. All study activities took place at the Infectious Diseases Institute, an adult outpatient HIV prevention care and treatment clinic in Kampala, Uganda. We conducted a randomized controlled trial in which intervention and control index participants were enrolled in 7 cohorts of about 30 participants each [36]. A blocked 1:1 randomization design was used with randomly alternating blocks of 2, 4, and 6, with stratification by gender. Between January 24, 2022 and February 21, 2023, PLHIV (index participants) were randomly assigned to receive the 8-session *Game Changers* intervention or to a no-intervention control group. Control group participants received usual care for HIV at the same clinic as intervention participants. Each enrolled index participant was asked to refer up to seven social network members to the study (hereafter referred to as “enrolled” alter participants), to assess whether the effects of *Game Changers* extended to social network members (who did not directly participate in intervention sessions). (The number of enrolled alters per index was based on the need to balance interviewing a sufficient sample of alters to be representative of the index’s network, while also taking into account prior social network research on chain referral of alters in stigmatized populations [37].) At baseline and at months 6, 12, and 18 post-baseline, both index and alter participants completed assessments. To cover transportation costs, participants were given 30,000 Ugandan Shillings (UGX; ~ 8USD) per assessment and intervention session attended. All participants provided written informed consent.

The study team engaged with two community expert groups throughout the study regarding study and intervention design and interpretation of results: the Infectious Diseases Institute community advisory board (CAB) and the National Forum of People Living with HIV/AIDS Networks in Uganda (NAFOPHANU).

### Intervention Description

The *Game Changers* intervention structure and content was developed by Ugandans in partnership with community stakeholders and U.S.-based researchers, and was based on prior empirical research in Uganda as well as stakeholders’ priorities and needs [12, 18, 19, 34]. The 8 weekly *Game Changers* sessions lasted about 2.5 h each and were conducted in Luganda (a commonly spoken local language). Session 1 introduced the program, defined internalized stigma, and discussed strategies for coping with stigma (adaptive coping, self-compassion). Session 2 further focused on empathy, self-compassion, and HIV serostatus disclosure decision-making, in order to foster self-compassion and establish comfort with disclosure and discussing HIV, prior to conducting prevention advocacy. Session 3 focused on HIV facts and myths and positive living with HIV: facilitators presented accurate HIV information and addressed common HIV misconceptions, and also discussed how PLHIV who model positive behaviors in their own life are more credible advocates. Participants were given information about HIV testing, condom use, and PrEP for discussion with alters who were HIV-negative or of unknown status; about condom use and antiretroviral therapy adherence to discuss with alters who were living with HIV; and about myths about HIV transmission (e.g., lack of risk of transmission through sharing utensils) for discussion with all alters, as needed. Participants were told practical, tangible information to convey to alters about how to access HIV prevention and treatment services (e.g., where to get tested, at no cost). Session 4 helped participants to understand how their personal social network can be used to promote HIV prevention; participants listed and mapped up to 20 close alters in their own social network (and strategically positioned alters), and identified alters to whom they disclosed and with whom they would like to do prevention advocacy. From the list of alters to whom they had not disclosed, participants were asked to think about the pros and cons of disclosure to each, and to consider disclosing if they wished to do prevention advocacy with them. Sessions 5–7 trained participants on effective prevention advocacy skills and strategies (teaching moments, open-ended questions, rephrasing) using role plays for practice with alters who might be in need of prevention advocacy (e.g., alters who discussed not using condoms, had multiple partners, showed signs of illness, and expressed belief in misconceptions about HIV). In Session 8, participants continued to practice prevention advocacy using role plays, discussed their experience with intervention participation, and affirmed their motivation and commitment to continuing prevention advocacy, and to draw on social support as needed; participants also received a graduation certificate in the last session. In all sessions, group interaction and sharing of personal experiences was encouraged to build social support and solidarity, and for participants to model for and learn problem solving skills from each other. After Session 1, all sessions included take-home activities to enable participants to implement disclosure decision-making and prevention advocacy skills in their lives, and report back to the group, to model effective behaviors to others and receive any needed problem solving and guidance from their peers.

### Intervention Facilitation: Training and Fidelity

Each intervention cohort was led by two peer facilitators (one man and one woman) who were fluent in both Luganda and English. The facilitators were trained to facilitate *Game Changers* sessions by study team members who had developed the intervention, including a clinical psychologist, a social psychologist, and a public health researcher (all of whom had expertise in intervention development), and an anthropologist (with expertise in social network analysis). The 4-day training included group facilitation skills and role plays of intervention content.

Fidelity was monitored by two Ugandan study coordinators who observed, rated, and provided feedback on each session using a form to record whether key session elements were adequately covered (1, not at all; 2, somewhat; 3, completely). The coordinators agreed on ratings for 73% of 260 session segments that were double-coded. High fidelity was evidenced by high interrater agreement (70%) that session content was “completely covered” in the 260 double-coded session segments.

### Participants and Eligibility Criteria

A total of 210 index PLHIV participants were enrolled and randomized after completing the baseline assessment, and 599 of their alters participated. Index participants were recruited at the Infectious Diseases Institute clinic, where team members presented the study opportunity to clients waiting for HIV services. Index participants were eligible if they were (1) at least 18 years-old; (2) living with HIV; (3) in HIV care at the study clinic for at least one year (so that they were likely to be sufficiently medically stable and adjusted to their HIV diagnosis to do prevention advocacy and complete the study); and (4) spoke Luganda fluently. Individuals were excluded if they participated in the prior intervention pilot study (NCT03435497), exhibited significant cognitive impairment, or a spouse/partner or household member was already enrolled as an index participant. Alter participants were eligible to be enrolled if they (1) were referred by an index participant; (2) were at least 18 years-old; (3) knew the index participant’s HIV serostatus; (4) spoke Luganda or English fluently; and (5) had sufficiently stable health status to complete the study. They were excluded if they exhibited significant cognitive impairment.

### Assessment

At each time-point, index participants completed a survey and social network assessment, and alter participants completed a survey only using the program Network Canvas (open-source survey and social network assessment software). The social network assessment used a longitudinal personal, egocentric network approach in which index participants were asked to list first names and last initials of up to 20 individuals with whom they had contact in the past year (hereafter referred to as “named” alters, only a portion of whom were enrolled as participants) [38]. Specifically, following recommendations for prompts to enhance recall from prior research [39], participants were asked, “I’d like you to name 20 people who you know and who know you. These should be people you have had contact with sometime during the past year or so, either face-to-face, by phone, mail, or email. They can be family members, friends, or people in your community (like neighbors, storekeepers, ministers, etc.). You can also think about people involved in providing you with HIV care or support, such as doctors, nurses, case managers, adherence buddies, and counselors, as well as other people with HIV. Let’s start by naming the people who are most important to you, and work outward toward people who have been less important. You can name any adults, no matter who they are or where they live, as long as they are important to you.”

At each follow-up, after participants named up to 20 alters, and their named alter list was compared to their list from the previous assessment; for any alters named in the prior but not current assessment, index participants were asked why they did not name the alter again, and whether they would like to add the alter to the current list of named alters. All assessment timepoints included the measures listed below. Internal reliability statistics for scales as measured at baseline are shown. All measures were translated into Luganda.

#### Primary Outcomes: Alter HIV Prevention Behaviors

Alter participants who were not living with HIV were asked whether they received *HIV testing* in the past 6 months and if so, their most recent test date and result. At baseline, all 392 alters who reported not living with HIV were asked about HIV testing; if alters reported an HIV-positive diagnosis at follow-up but reported being HIV-negative on the prior survey, their response to the self-reported HIV testing item at follow-up was imputed as having been tested prior to the survey.

Participants were asked about frequency of condom use in the past 6 months with main and casual sexual partners in separate items, as applicable, on 5-point Likert scales (1, never; 2, rarely; 3, sometimes; 4, often; 5, always). Participants also reported the number of casual partners they had in the past 6 months, and of those times (if any), the number of times they used condoms. From these items, we derived two measures of condom use: a dichotomous strict definition of ***consistent condom use*** operationalized as “all of the time” (limited HIV risk) vs “less than all of the time” (any HIV risk) across both main and casual partners (based on whether “always” was reported on the applicable 5-point frequency scales, and use of condoms for 100% of sexual intercourse occasions with casual partners in the past 6 months); and a continuous measure of perceived past-6 month *frequency of condom use* across both main and casual partners (mean of the two 5-point frequency scales if both types of partners were reported). This perceived frequency measure allowed for a harm reduction approach (i.e., greater use of condoms, even if not all of the time, can lower HIV risk).

Note that the measure of consistent (100%) condom use, the dichotomous variable, was classified as “no” for participants who reported sexual intercourse in the past 6 months but did not answer the condom use question(s) (n = 7 at baseline). Participants who reported not having a main or casual partner (n = 166) or not having sex in the past 6 months (n = 3), were scored as “missing” for both condom use variables (n = 169). One additional participant reported having a casual partner but did not respond to the frequency item; thus, data on the extent of condom use variable was missing for n = 170.

#### Secondary Outcomes: Index Participant HIV Prevention Advocacy and Psychosocial Outcomes

In the social network assessment, index participants were asked whether they engaged in *HIV prevention advocacy* with each named alter (i.e., each alter named by the index participant in the social network assessment) in the past 3 months, with separate questions for each relevant HIV prevention behavior (condom use for all alters, HIV testing and PrEP for HIV-negative alters, and HIV care and ART use for alters with HIV). In parallel questions, alter participants were asked whether they discussed each relevant HIV prevention behavior with the index in the past 3 months. We derived a dichotomous dyadic measure of whether both the index and alter reported that the index participant conducted any HIV prevention advocacy with the alter. Lastly, we derived a social network measure of proportion of named alters with whom the index conducted any prevention advocacy (i.e., any of the relevant specific HIV prevention behaviors were discussed) in the past 3 months.

*HIV prevention advocacy self-efficacy* was assessed with a single item adapted from prior research [40]: “How confident are you that you can start a conversation about HIV with people you know, like with your family members and friends?” from 0–10, with 10 indicating high self-efficacy (0, cannot do at all to 10, certain I can do). *Internalized HIV stigma* was assessed with mean item values on the 6-item Internalized AIDS-Related Stigma Scale (alpha = 0.80) [41]. *HIV serostatus disclosure* was assessed on the social network interview, with the index participant reporting on whether each named alter knew the HIV serostatus of the index; we derived a measure indicating the proportion of alters who knew the index’s HIV serostatus. *HIV knowledge* was assessed with 5 items from a validated scale for PLHIV (e.g., “An HIV positive individual does not need to take medication for HIV everyday if they do not have any symptoms”) [42], 2 items from the Uganda Demographic and Health Survey (e.g., “A person can get HIV by sharing food with a person who has HIV/AIDS”) [5], and 6 items that were tailored to the intervention content and cultural context (e.g., “Spiritual healing is effective against HIV”). Response options were “yes,” no,” and “don’t know,” from which a sum of correct responses was derived.

#### Potential Covariates

*Socio-demographic characteristics* were assessed for both index and alter participants, and included age, sex, education level, occupation, monthly household expenditure, marital/relationship status, and HIV diagnosis date (if applicable).

### Statistical Analysis

We hypothesized that index PLHIV in the intervention group would show greater prevention advocacy, greater self-efficacy for prevention advocacy, higher HIV knowledge, more serostatus disclosure in their social networks, and lower internalized stigma at follow-up, compared to index PLHIV in the control group, and that their alters (who also were enrolled in the study) would show increased HIV testing and condom use (as a result of the intervention’s effects on index participants). Intention-to-treat multivariate regression models were used to test intervention efficacy, comparing outcomes between the intervention and control arms at all follow-up time points; logistic regressions were used for binary outcomes and linear regressions were used for continuous outcomes. Descriptively, we present the means by group at each follow up. For regression analysis, all follow-up times are pooled. Using a ‘tall’ dataset with stacked records for all follow-up time points for each individual, repeated measures linear and logistic regression models included fixed effects for the intervention condition, the baseline value of the outcome, and covariates, if any. Models also included a random effect for the index participant, and for outcomes at the alter level, models additionally included a random effect for alter nested within index participant. The regressions included all individuals with values for the outcome at baseline and at least one follow-up time-point, with each participant contributing up to three follow-up responses (from the 6-, 12-, or 18-month assessments).

In post-hoc analyses that followed guidance for exploring intervention dosage effects [43], and following our conceptual model linking index prevention advocacy directly with alter behaviors, we conducted within-intervention group analyses with data from alters of index participants in the intervention condition to examine whether alters who were exposed to any prevention advocacy at any follow-up time point were more likely to change their condom use and HIV testing behavior than alters who were not exposed to any prevention advocacy at follow-up. Moreover, as a sensitivity analysis, we conducted a per-protocol analysis [44], subsetting the treated group to those with who completed all sessions (n = 82) to assess maximum treatment efficacy (i.e., to examine intervention effects when index participants received the full dose of all intervention sessions).

Covariates were defined as any socio-demographic or relational variable that significantly differed by intervention arm (Table 1). Outcomes were never missing among applicable participants. Maximum likelihood assumes missingness at random [45]; therefore, we did not impute entirely missing surveys. Participants were included in the model predicting that outcome if they had a value for the outcome at baseline and at any follow-up. We controlled for multiple comparisons using a Bonferroni correction, by dividing alpha = 0.05 by the number of tests within each set (alter primary outcomes, index secondary prevention advocacy outcomes, other index secondary outcomes).

**Table 1:** Index and Alter Participant Characteristics

	Intervention (n = 105)	Control (n = 105)	Overall (N = 210)
Index Participants (n = 210)			
Age in years M (SD)	40.7 (10.7)	39.3 (10.5)	40.0 (10.6)
Woman (%)	70.5	69.5	70.0
Married/In Relationship (%)	66.7	65.7	66.2
Education Level (%)			
Lower primary	11.9	7.7	9.8
Upper primary	31.7	26.9	29.3
Lower secondary	40.6	41.4	41.0
Upper secondary	5.0	9.6	7.3
University	8.9	11.5	10.2
Other	2.0	2.9	2.4
Occupation (%)			
Farmer	5.7	8.6	7.1
Salaried employee	13.3	21.0	17.1
Business/vendor	67.6	53.3	60.5
Casual worker	3.8	2.9	3.3
Other	9.5	14.3	11.9
Estimated average household monthly expenditure (%)			
< 10,000 Ugandan Shillings (UGX)	0.0	0.0	0.0
10,000–30,000 UGX	0.0	1.0	0.5
30,001–50,000 UGX	1.0	2.9	1.9
50,001–100,000 UGX	2.9	2.9	2.9
100,001–500,000 UGX	72.1	70.2	71.2
> 500,000 UGX	24.0	23.1	23.6
Length of Time since HIV Diagnosis in years [M (SD)]	10.8 (6.1)	11.1 (5.2)	11.0 (5.6)
Alter Participants (n = 599)			
Age in years [M (SD)]	36.6 (12.3)	38.2 (12.4)	37.4 (12.4)
Woman (%)	68.8	63.3	66.3
Married/In Relationship (%)	75.0	78.6	76.6
Education Level (%)			
Lower primary	10.1	8.6	9.4
Upper primary	24.3	22.7	23.6
Lower secondary	36.0	33.8	35.0
Upper secondary	12.3	10.8	11.6
University	13.6	16.4	14.9
Other	3.8	7.8	5.6
Occupation (%)			
Farmer	9.0	5.1	7.2
Salaried employee	17.7	19.6	18.6
Business/vendor	45.0	53.8	49.1
Casual worker	5.6	4.7	5.2
Other	22.7	16.7	19.9
Estimated average household monthly expenditure (%)			
< 10,000 Ugandan Shillings (UGX)	0.6	0.4	0.5
10,000–30,000 UGX	0.9	0.0	0.5
30,001–50,000 UGX	1.2	0.4	0.9
50,001–100,000 UGX	4.4	3.7	4.1
100,001–500,000 UGX	66.4	55.0	61.2
> 500,000 UGX	26.5	40.5	32.9
Living with HIV (%)	33.2	38.9	35.8

There was a statistically significant difference for alter participant monthly expenditure (p < .001 per t-test). No other differences were significant at the p = .05 level. UGX = Ugandan Shillings. The study population was relatively similar to the clinic population from which participants were sampled: Both the study and clinic population had been living with HIV an average of 11 years, and the study sample’s average age was 40 years-old (vs. 46 years-old in the clinic population). Approximately 70% of the study sample was women (compared to a slightly lower 63% women in the clinic population)

## Results

### Participant Flow and Characteristics

**Fig. 1:**
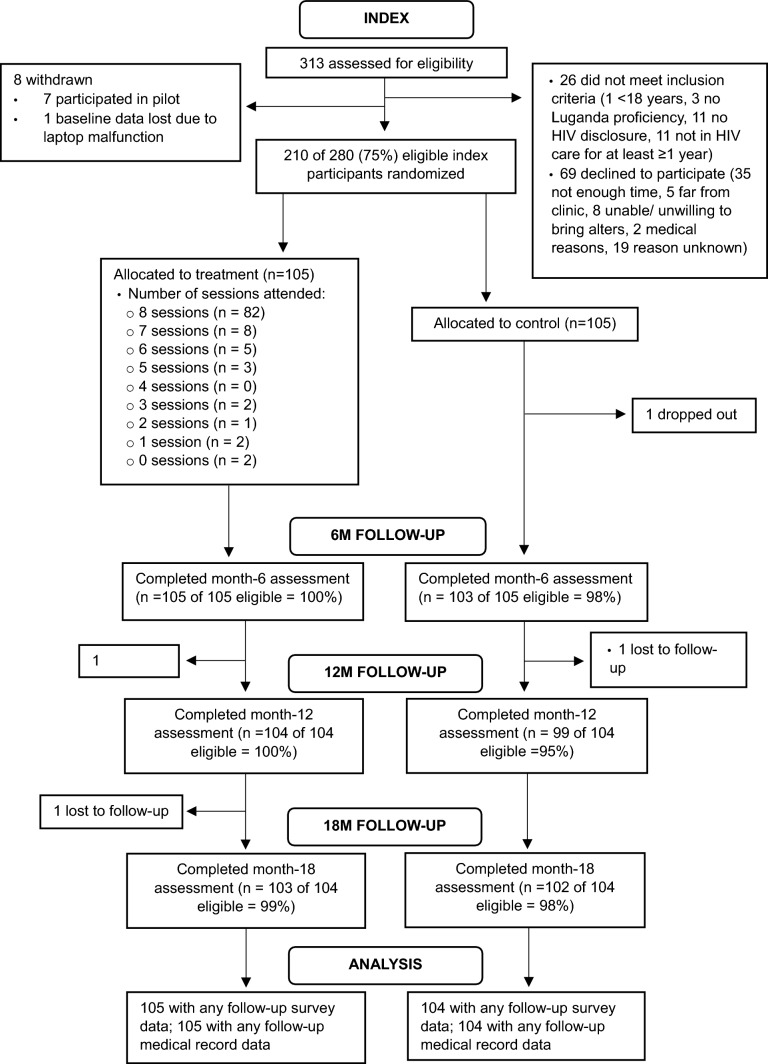
Index CONSORT Diagram

As shown in the index participant CONSORT diagram (Fig. 1), 313 index PLHIV were screened for eligibility, of whom 26 were ineligible and 69 declined to participate (e.g., too busy). An additional 8 participants were withdrawn after randomization: seven were found to be ineligible due to participation in the pilot study, and data were lost for one participant due to laptop malfunction. Thus, 210 eligible index participants completed the baseline assessment and were randomized (105 intervention, 105 control). Nearly all (99%) completed the 6-month follow-up (of 210 eligible), 98% (of 208 eligible) completed the 12-month follow-up; and 99% (of 208 eligible) completed the 18-month follow-up. The analysis sample included all 209 participants (105 intervention, 104 control) who had survey data from at least one follow-up.

**Fig. 2:**
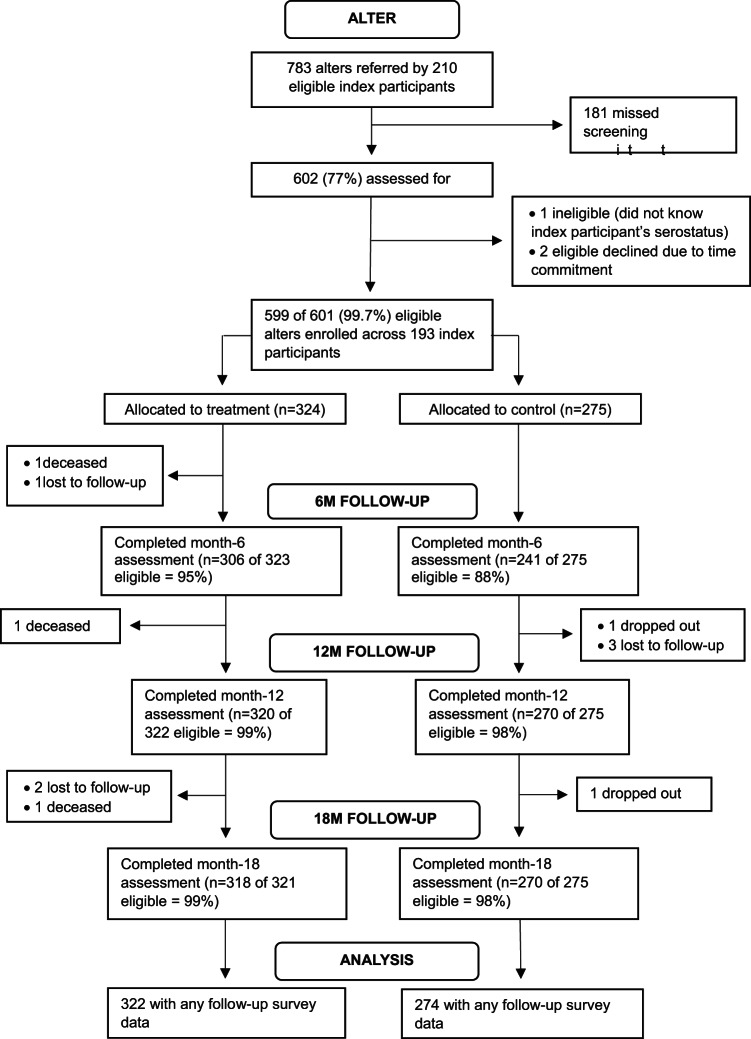
Alter CONSORT Diagram. Duplicate data from 2 alter participants were removed from the study, because they were recruited, enrolled, and completed the baseline assessment twice. Data from an additional 76 alter participants were removed because data from 50 participants were collected without adequate consent or eligibility assessment, and data from 26 participants were determined to be fabricated or possibly fabricated. The inadequate consents and data fabrication were conducted on the part of a single data collector. Once these incidents were detected, a full investigation was conducted by the Research Integrity Committee of the Infectious Diseases Institute and reported to the US Office of Research Integrity. All data presented in this article were validated through direct check-ins with participants

As shown in the alter participant CONSORT diagram (Fig. 2), 783 alters were referred across 210 eligible index PLHIV participants; of these, 602 alters (77%) were screened and 599 alters (77%) were enrolled across 193 index PLHIV participants (324 intervention, 275 control). Of those screened, only one alter was ineligible and only two eligible alters refused. Nearly all (98%) of 598 eligible alters completed the 6-month follow-up, 99% of 597 eligible alters completed the 12-month follow-up, and 99% of 596 eligible alters completed the 18-month follow-up. The analysis sample included 596 alters with any follow-up data (322 intervention, 274 control). As only one index participant and three alter participants were excluded from the analysis (due to drop-out), we did not examine baseline characteristics of those included in the analysis compared to those excluded.

Seven sets of intervention sessions were conducted between March 16, 2022, and April 5, 2023. Of the 105 index participants assigned to the intervention, 98% (n = 103) attended at least one session, and 78% (n = 82) attended all sessions; only two participants did not attend any sessions.

Table 1 shows baseline index and alter socio-demographic and index-alter relationship characteristics, among participants in the final analysis sample. The intervention and control groups did not significantly differ on any baseline characteristic apart from alter participant monthly expenditure, which was lower among intervention vs. control participants (p < 0.001 per t-test) and therefore included as a covariate in all alter-level regressions. Alter monthly household expenditure was missing for nine alters (1.5%) and was imputed using the overall mean.

### Intervention Effects on Index Participant Prevention Advocacy

As shown in Table 2, the repeated-measures regression models indicated that, as hypothesized, intervention index participants were more likely to engage in any HIV prevention advocacy with enrolled alters (a small-to-medium effect size of 0.3), and engaged in any HIV prevention advocacy with a greater percentage of alters they named in the social network assessment (a small-to-medium effect size of 0.3) across the follow-up timepoints compared to their counterparts in the control group. Both effects were significant with a Bonferroni correction of alpha = 0.025.

**Table 2:** Effects of *Game Changers* on Index Participant HIV Prevention Advocacy (PA) at the Dyadic and Social Network Levels

	Baseline M (SD) or %		6-Month Follow-Up M (SD) or %		12-Month Follow-Up M (SD) or %		18-Follow-Up M (SD) or %		Repeated Measures Intervention Efficacy Tests ^^^	
	Intervention	Control	Intervention	Control	Intervention	Control	Intervention	Control	b (SE) or OR (95% CI), p	Effect Size
Dyadic level										
Received any PA from index (enrolled alter)* (n = 570)	36.4%	34.3%	58.9%	41.4%	56.9%	44.0%	64.5%	54.5%	OR (CI) = 1.70 (1.16–2.51), p = .007	.29
Social Network Level										
Percentage of alters given any PA by index (named alters) (n = 209)	29.45 (25.35)	31.47 (27.81)	55.18 (28.27)	30.13 (23.96)	53.13 (28.74)	37.70 (28.19)	56.91 (26.15)	44.86 (26.10)	b (SE) = 17.89 (3.05), p < .001	.31

* Presence of PA reported by both the index and alter

^ Means are presented at each follow up for descriptive purposes. For statistical testing, all follow-up times are pooled (with standard errors adjusted for clustering)

Individual-level (alter report) and dyadic level regression models included baseline alter monthly expenditure (which significantly varied by intervention condition) as a covariate

### Intervention Effects on Index Psychosocial Outcomes

Consistent with hypotheses, intervention index participants reported lower levels of internalized HIV stigma (a small effect size of 0.2), more serostatus disclosure (a small effect size of 0.1), greater HIV prevention advocacy self-efficacy (a small-to-medium effect size of 0.3), and greater levels of HIV knowledge (a small effect size of 0.2) than did control index participants at follow-up, compared to baseline (Table 3). With a Bonferroni correction, using an alpha of 0.0125, all of the index psychosocial outcomes were significant.

**Table 3:** Effects of *Game Changers* on Index HIV-Status Disclosure, Internalized HIV Stigma, and HIV Prevention Advocacy Self-Efficacy

	Baseline M (SD)		6-Month Follow-Up M (SD)		12-Month Follow-Up M (SD)		18-Follow-Up M (SD)		Repeated Measures Intervention Efficacy Tests ^^^	
	Intervention	Control	Intervention	Control	Intervention	Control	Intervention	Control	b (SE), p	Effect Size
Internalized HIV stigma (n = 209)	2.37 (1.16)	2.31 (0.94)	1.61 (0.68)	2.15 (1.02)	1.66 (0.90)	2.10 (1.11)	1.71 (0.84)	2.06 (1.00)	−0.46 (0.09), p < .001	-.24
Disclosure (mean percentage of named alters who know index serostatus) (n = 209)	53.89 (33.15)	49.00 (31.34)	58.34 (26.63)	48.09 (28.58)	57.11 (28.68)	50.43 (28.70)	60.76 (28.38)	50.40 (26.51)	6.86 (2.75), p = .01	.12
HIV prevention advocacy self-efficacy (n = 209)	7.01 (2.22)	7.28 (2.11)	8.41 (2.10)	7.25 (2.27)	8.50 (2.00)	7.22 (2.41)	8.46 (1.92)	7.25 (2.45)	1.28 (0.21), p < .001	.28
HIV knowledge (n = 209)	10.70 (1.40)	10.45 (1.28)	11.62 (1.27)	11.07 (1.12)	11.90 (1.16)	11.38 (1.15)	12.04 (1.04)	11.59 (1.22)	0.45 (0.12), p < .001	.19

^ Means are presented at each follow up for descriptive purposes. For statistical testing, all follow-up times are pooled (with standard errors adjusted for clustering)

### Intervention Effects on Primary Alter Behavior Outcomes

As shown in Table 4, repeated-measures regression analyses indicated no significant overall hypothesized intervention effects on alter HIV testing and frequency of condom use at follow-up. Consistent condom use among alters was marginally higher at follow-up compared to baseline, with a small-to-medium (0.3) effect size.

**Table 4:** Effects of *Game Changers* on Alter HIV Testing and Condom Use

	Baseline % or M (SD)		6-Month Follow-Up % or M (SD)		12-Month Follow-Up % or M (SD)		18-Follow-Up % or M (SD)		Repeated Measures Intervention Efficacy Tests ^^^	
	Intervention	Control	Intervention	Control	Intervention	Control	Intervention	Control	OR (95% CI) or b (SE), p	Effect Size
HIV testing, past 6 months (n = 389)^a^	43.6%	47.4%	72.0%	72.2%	68.2%	71.3%	77.0%	78.3%	0.97 (0.66–1.44), p = .89	-.02
Consistent condom use (100%), past 6 months (n = 408)^b^	13.9%	14.5%	12.7%	13.5%	15.5%	11.7%	16.2%	8.1%	1.56 (0.92–2.63), p = .097	.25
Average extent of condom use (1–5), past 6 months (n = 406)^b^	2.03 (1.52)	2.02 (1.52)	2.07 (1.50)	2.08 (1.51)	2.21 (1.56)	1.98 (1.45)	2.19 (1.58)	1.90 (1.39)	0.12 (0.11), p = .27	.04

^ Means are presented at each follow up for descriptive purposes. For statistical testing, all follow-up times are pooled (with standard errors adjusted for clustering)

All regression models included as a covariate baseline alter monthly expenditure (which significantly varied by intervention condition)

^a^Restricted to alters without HIV

^b^Restricted to alters with a main or casual partner

Post-hoc exploratory within-intervention analyses indicated significant effects of index participant engagement in any prevention advocacy with the alter (as reported by both the index and alter) on the primary alter behavior outcomes, controlling for alter monthly expenditure. Alters of intervention index participants who received any prevention advocacy from the index at follow-up were more likely to report recent (past 6 months) HIV testing [OR (95% CI) = 1.78 (1.11–2.86), p = 0.02], and more frequent condom use [b (SE) = 0.25 (0.12), p = 0.04] at follow-up, compared to alters who did not have any advocacy discussions with the index participant; the groups did not differ on rates of consistent condom use [OR (95% CI) = 1.55 (0.80–3.02), p = 0.20].

The findings and conclusions of the per-protocol analyses were the same as the intention-to-treat analyses; thus, participants who were fully compliant with the intervention protocol appeared to be comparable to participants who did not complete the intervention (and non-compliance likely did not systematically bias the results). Specifically, the per-protocol analysis showed effects on index participants for prevention advocacy with enrolled alters [OR (CI) = 1.61 (1.06–2.45), p = 0.03], engagement in prevention advocacy with a greater percentage of named alters [b (se) = 18.25 (3.31), p < 0.001], lower internalized stigma [b (se) = −0.46 (0.09), p < 0.001], and higher serostatus disclosure [b (se) = 6.58 (2.91), p = 0.02], HIV prevention advocacy self-efficacy [b (se) = 1.35 (0.22), p < 0.001], and HIV knowledge [b (se) = 0.47 (0.13), p < 0.001].

## Discussion

Consistent with our pilot [34], our randomized controlled trial of the *Game Changers* intervention demonstrated robust effects on the index PLHIV who directly received the peer advocacy training intervention, including reduced internalized stigma, increased serostatus disclosure, and engagement in HIV prevention advocacy—variables found to be associated with HIV prevention behaviors in prior research [11, 46, 47]. Our data did not support a direct effect of the intervention on the HIV prevention behaviors of the social network members (alters) of the index participants, despite its effects on increased advocacy within these social networks. However, when HIV prevention advocacy occurred between the index and specific alters (as tested in post-hoc analyses), this advocacy had a significant effect on alters’ increased HIV testing and condom use. These results demonstrate the effectiveness of training PLHIV to increase discussions about HIV throughout social networks and its benefits for both PLHIV who receive the training, and their social network members with whom they engage in HIV prevention advocacy. Further, our findings are in line with prior research showing that anti-stigma interventions that increase knowledge and awareness about HIV can decreased internalized stigma [24], and that social network interventions that train people on prevention advocacy can improve HIV prevention behaviors [20].

Our intervention used a staged approach, addressing internalized stigma, decision-making related to HIV serostatus disclosure (i.e., weighing risks and benefits to disclosure), and HIV knowledge and misconceptions in initial sessions, prior to teaching PLHIV skills to engage social network members in prevention advocacy. The initial sessions were intended to build a foundation for increased skills and confidence to engage in advocacy. The increased HIV prevention advocacy conducted by PLHIV within their social networks (and with enrolled alters specifically) was in turn expected to lead to behavior change and increased alter HIV testing and condom use. We did not find intervention effects on social network members’ self-reported HIV testing and condom use. However, in post-hoc analyses, consistent with our staged intervention approach, alters with whom PLHIV engaged in prevention advocacy evidenced a greater likelihood of recent HIV testing and condom use, compared to alters who did not receive advocacy. Thus, alter behavior change may be contingent on index behavior change (i.e., increased engagement in advocacy)—and alter behavior change may show delayed, stronger effects over a longer time-period than assessed in the present study. Moreover, the modest effect sizes observed for alter behaviors and the lack of alter behavior change effects in intention-to-treat analyses may have been due to unmeasured moderating factors (e.g., alters’ existing attitudes that are resistant to change, or social influence from peers or partners outside of the intervention’s reach). While plausible, it is not guaranteed that randomization led to balance in these unmeasured factors in the intervention and control groups, especially since index participants were randomized, not alters. Future research could better discern factors that determine the success of advocacy on promoting alters’ behavior change.

Limitations include reliance on self-reported HIV prevention behaviors, which can be biased by social desirability, a possible selection bias for both index and alter participants, and the relatively small effect sizes that were observed in the intention-to-treat analyses. Self-reported HIV testing was not confirmed with medical records, which would not have been feasible to collect from a range of different clinics and testing events in Uganda. Index PLHIV may have been a select sample of individuals who were motivated to participate in an HIV prevention study in which they might receive advocacy training. Our within-intervention analyses cannot distinguish between intervention effects versus index participants’ motivation to conduct prevention advocacy, which can vary by individual difference factors such as motivation for advocacy and family history of HIV [48–50]. Moreover, alter recruitment was limited to individuals to whom index participants disclosed their serostatus, referred to the study, and could be reached. It is possible that this subset of potential alters had increased motivation to engage in HIV protective behaviors. Recruiting a wider cross-section of alters or a subset of alters at higher risk for HIV may have improved our ability to detect effects; however, the need to limit risks to the index participant, and to produce findings that were as generalizable as possible, contributed to the decision to limit the alter subsample. Additionally, we may not have enrolled enough alters, or the proportion of alters for whom HIV testing was relevant may have not been sufficiently high to be able to detect effects. We also did not control for attention and instead randomized to a usual care control group, because the target of the intervention was alter behavior change, and alters did not attend intervention sessions; thus, we do not believe that changes in alter behavior could be attributed to index PLHIVs’ attention in intervention sessions.

## Conclusion

Since our promising pilot [34], other research has applied the *Game Changers* model of peer advocacy to promote health behavior change to cervical cancer prevention, and adapted aspects of the model for PrEP promotion [51, 52]. Future research could explore mediators and moderators of *Game Changers*’ effects, as well as gauge *Game Changers*’ effects on whole communities, examining whether external HIV stigma decreases and HIV prevention behavior consequently increases in a representative sample of community members over time. Research could also explore effects of the intervention on the social networks of alters, in terms of whether alters, and alters of alters, further disseminate HIV prevention information in their own social networks. Implementation research could be conducted to adapt and disseminate the intervention through online platforms, especially for youth and young adults. Overall, *Game Changers* represents a feasible social network intervention model that can be applied across health conditions.

## Data Availability

All authors ensure that the data support the published claims and comply with field standards.
